# Evolution of Minimum Mortality Temperature in Stockholm, Sweden, 1901–2009

**DOI:** 10.1289/ehp.1509692

**Published:** 2015-11-13

**Authors:** Daniel Oudin Åström, Andreas Tornevi, Kristie L. Ebi, Joacim Rocklöv, Bertil Forsberg

**Affiliations:** 1 Division of Occupational and Environmental Medicine and; 2 Division of Epidemiology and Global Health, Department of Public Health and Clinical Medicine, Umeå University, Umeå, Sweden

## Abstract

**Background:**

The mortality impacts of hot and cold temperatures have been thoroughly documented, with most locations reporting a U-shaped relationship with a minimum mortality temperature (MMT) at which mortality is lowest. How MMT may have evolved over previous decades as the global mean surface temperature has increased has not been thoroughly explored.

**Objective:**

We used observations of daily mean temperatures to investigate whether MMT changed in Stockholm, Sweden, from the beginning of the 20th century until 2009.

**Methods:**

Daily mortality and temperature data for the period 1901–2009 in Stockholm, Sweden, were used to model the temperature–mortality relationship. We estimated MMT using distributed lag nonlinear Poisson regression models considering lags up to 21 days of daily mean temperature as the exposure variable. To avoid large influences on the MMT from intra- and interannual climatic variability, we estimated MMT based on 30-year periods. Furthermore, we investigated whether there were trends in the absolute value of the MMT and in the relative value of the MMT (the corresponding percentile of the same-day temperature distribution) over the study period.

**Results:**

Our findings suggest that both the absolute MMT and the relative MMT increased in Stockholm, Sweden, over the course of the 20th century.

**Conclusions:**

The increase in the MMT over the course of the 20th century suggests autonomous adaptation within the context of the large epidemiological, demographical, and societal changes that occurred. Whether the rate of increase will be sustained with climate change is an open question.

**Citation:**

Oudin Åström D, Tornevi A, Ebi KL, Rocklöv J, Forsberg B. 2016. Evolution of minimum mortality temperature in Stockholm, Sweden, 1901–2009. Environ Health Perspect 124:740–744; http://dx.doi.org/10.1289/ehp.1509692

## Introduction

Extreme ambient temperatures, be they hot or cold, are known to cause negative effects on human health (Anderson and Bell 2009; Kovats and Hajat 2008). The temperature-mortality relationship is often described as a J- or U-shaped curve, with a temperature at which mortality is at a minimum, known as the minimum mortality temperature (MMT) (Baccini et al. 2008; Curriero 2002; Guo et al. 2014; Leone et al. 2013; McMichael et al. 2008; Medina-Ramón and Schwartz 2007). This minimum temperature varies greatly across countries and regions, ranging from a daily mean temperature of 10–12°C in Scandinavian countries (Nafstad et al. 2001; Näyhä 2007) to 27°C in Miami, Florida (Curriero 2002).

If the shape of the temperature distribution remains the same in a warmer climate, albeit with a higher mean temperature, and if MMT is a stationary measure over time, then one would anticipate, all other factors being equal, that as the proportions of temperatures above and below the MMT threshold shift, cold-related mortality would decrease and heat-related mortality would increase. Evidence shows that temperature distributions are shifting to more days with warmer temperatures and fewer days with colder temperatures (Field et al. 2012). Mean daily temperatures increased over the 20th century in Stockholm, Sweden (SMHI 2013), and temperatures are projected to increase further (Nikulin et al. 2011).

Adaptation could offset some of the mortality from higher temperatures by shifting the MMT to higher values. To support this hypothesis, the reduction of short-term mortality to regional hot-temperature extremes has been observed in Stockholm, Sweden, over time (Åström et al. 2013). A few other studies have documented the extent to which adaptation and acclimatization occurred over the 20th century (Carson et al. 2006; Ekamper et al. 2009; Petkova et al. 2014). There is speculation that, as a consequence of increasing temperatures, climate change might itself be a factor for acclimatization both on an individual level (physical acclimatization and use of air conditioners), as well as on a societal level (urbanization) (Parry et al. 2007). There is also speculation that milder temperatures will reduce excess winter mortality (McMichael and Lindgren 2011), although the evidence base is limited.

The MMT could be affected by changing demography, in particular an increased proportion of elderly in the population. The expected rise in the number of elderly and other potentially vulnerable groups, both in absolute numbers and as a proportion of the population, could increase the severity of the impact of temperature extremes on human health (Sierra et al. 2009) because the elderly and the chronically ill are more sensitive to temperature extremes than other groups within the general population (Anderson and Bell 2009; Basu 2009; Oudin Åström et al. 2011).

The aim of the present study was to investigate whether the MMT changed in Stockholm, Sweden, between the beginning of the 20th century and 2009. To our knowledge, this is the first study of the evolution of MMT to use daily mortality and temperature data spanning more than a century, increasing the power to test for trends.

## Materials and Methods

We collected daily mortality and temperature data for the period 1901 to 2009 for Stockholm County, Sweden. The mortality data were deaths from all causes (Sveriges släktforskarförbund 2010). The meteorological data were daily mean temperatures provided by the Swedish Meteorological and Hydrological Institute (SMHI) (SMHI 2013). Temperature data have been recorded in Stockholm at almost exactly the same location since 1756.

### Statistical Methods

The evolution of the MMT over time was modeled using a time series approach in which the daily counts of mortality were assumed to follow an overdispersed Poisson distribution. The temperature–mortality relationships were modeled using a distributed lag nonlinear model (DLNM) with a lagged period of 21 days to account for the immediate effects of heat and for short-term mortality displacement. Twenty-one days should be sufficient to capture delayed effects of cold (Gasparrini et al. 2015).

To avoid large influences from intra- and interannual climatic variability as manifested by an unusually large number of temperature extremes in some decades, we estimated the MMT for 30-year periods containing daily observations for mortality and temperature. The smoothed evolution of MMT, explaining broad patterns of change, was examined by repeating the model stepwise across the century and including 30 years of data in each model while changing the time period of study by 1 year at a time. Thus, the first data point created to calculate the MMT corresponded to observations for the period 1901–1930, the second for the period 1902–1931, and so forth until the last data point, which corresponded to the period 1980–2009. The model was run 80 times, and the MMT was extracted for each period.

MMTs for Stockholm were derived using the following model for each of the different periods investigated:

Y*_t_* ~ Poisson(μ*_t_*)

log(μ*_t_*) = α + βT*t_i_* + weekday*t* + holiday*t* + NS(trend, df = 8 per year), [1]

where Y*_t_* is the number of deaths occurring in Stockholm County on day *t*, α represents the intercept, and βT*t_i_* is a vector of *i* coefficients representing the nonlinear and lagged effects of mean temperature on day *t*, modeled as a quadratic B-spline with two equally spaced knots for temperature and as a natural cubic spline with three equally spaced knots (log scale) for the 21-day lag period, respectively. Weekday is a categorical variable for day of the week, and holiday is a binary variable indicating Swedish public holidays. NS(trend) is a natural cubic spline with 8 degrees of freedom per year to take into account variability in mortality owing to seasonality and longer-term time trends.

Sensitivity analyses were performed using 10 degrees of freedom for the time trend.

In addition, for each 30-year period, we estimated the relative value of the MMT, defined as the percentile of the same-day (lag0) temperature distribution corresponding to the absolute value of the MMT. We performed this procedure to investigate whether changes in the MMT over time were accompanied by changes in its location within the overall temperature distribution.

The smoothed evolution of the MMT over time did not have more than three independent time periods of observation at the same time. To allow valid inferences regarding the evolution of MMT to be made, we used data from nonoverlapping, and therefore independent, time periods of 10 and 20 years of data. The 10-year periods investigated were 1901–1909, 1910–1919, and so forth, up until 2000–2009. The 20-year periods investigated were 1910–1929, 1930–1949, 1950–1969, 1970–1989, and 1990–2009. Using the model described above, we derived MMTs for the independent time periods. We then investigated if a trend over time could be detected using linear regression models with either the absolute MMT or the relative MMT as the dependent variable, and time as the independent variable.

SAS v.9.2 software (SAS Institute Inc., Cary, NC) was used to create data sets and variables. R v.2.13.1, package DLNM, was used for statistical models and creation of outputs (R Core Team 2015).

## Results

**Figure 1:**
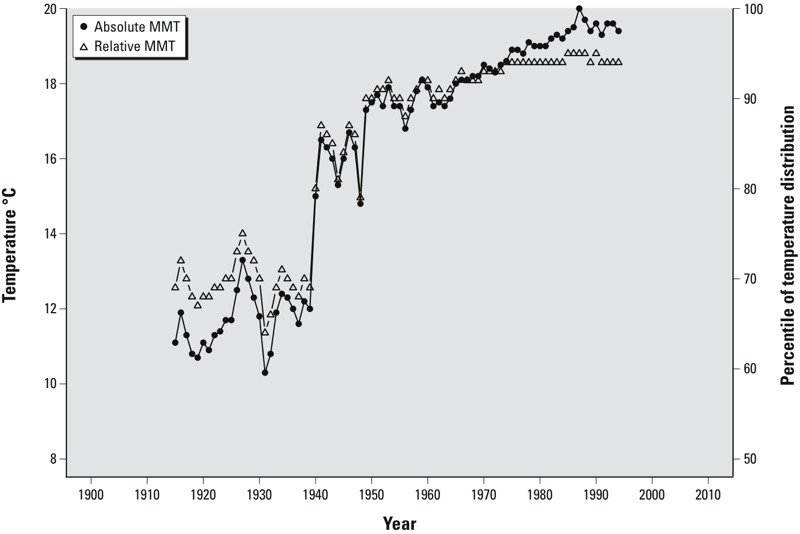
Estimates of minimum mortality temperature (MMT) during 1901–2009 in Stockholm, Sweden. Filled circles indicate the absolute value of the estimated MMT (°C), and open triangles indicate the corresponding estimate of the MMT as a percentage of the temperature distribution. Estimates were derived using distributed lag non-linear models of 30-year moving averages with a 21-day lag, adjusted for weekdays, holidays, and smoothed time trends (8 degrees of freedom per year). The smoothed evolution of the MMT was examined by repeating the model stepwise across the century while including 30 years of data in each model and changing the time period of study by 1 year at a time. Thus, the first data point created for calculating the MMT corresponds to observations for 1901–1930, which was centered around the year 1915; the second corresponds to observations for 1902–1931 (centered around the year 1916); and so forth until the last data point, which corresponds to 1980–2009.

We observed a trend over time for both the absolute MMT and the relative MMT (Figure 1). Before 1950, MMTs occurred at lower temperatures and were much more variable than later in the study period.

Throughout the 20th century, MMTs ranged from 10.3°C to 20.0°C (median = 17.4°C). Relative MMTs ranged from the 64th to the 95th percentile of the lag0 temperature distribution (median = 90th percentile).

Similar results were found in the sensitivity analyses, which used 10 degrees of freedom for the time trends (results not shown).

**Figure 2:**
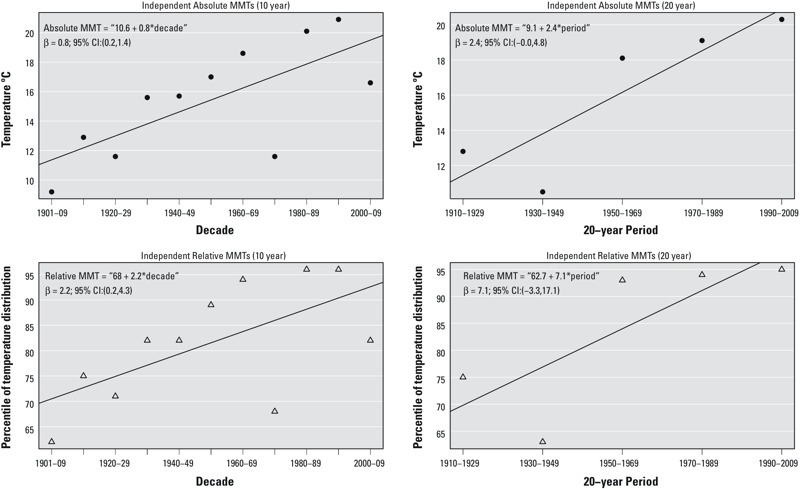
Independently estimated minimum mortality temperatures (MMTs) during 1901–2009 in Stockholm, Sweden. Filled circles indicate the absolute value of the estimated MMT (°C), and open triangles indicate the corresponding estimate of the MMT as a percentage of the temperature distribution. Estimates were derived using distributed lag non-linear models of independent 10- and 20-year periods with a 21-day lag, adjusted for weekdays, holidays, and smoothed time trends (8 degrees of freedom per year).

Estimates of MMTs for nonoverlapping 10- or 20-year periods were variable (Figure 2). We estimated an increasing trend for the absolute value of MMT over time (βtrend 10 years = 0.8; 95% CI: 0.2, 1.4; βtrend 20 years = 2.4; 95% CI: –0.0, 4.8). Relative MMT as a percentile of the overall distribution also increased over time when estimated for independent 10-year (βtrend 10 years = 2.2; 95% CI: 0.2, 4.3) and 20-year (βtrend 20 years = 7.1; 95% CI: –3.3, 17.5) periods.

## Discussion

We report a trend of increasing MMT over time in Stockholm, Sweden. Over the course of the 20th century, large changes occurred in Swedish society, yielding large benefits to public health. Housing standards improved significantly after the 1950s with the introduction of central heating, and sanitary conditions were improved. The epidemiological transition changed mortality patterns and delayed mortality to older ages (Omran 1971), resulting in increasing life expectancy at birth and a larger proportion of the population being elderly. For men and women, life expectancy at birth increased from approximately 53 and 56 years, respectively, during the early 1900s to approximately 79 and 83 years, respectively, in 2009 (Statistics Sweden 2015). The percentage of deaths taking place among people > 65 years old increased from approximately 30% during the 1900s and the 1910s to > 75% from the 1980s onwards. The uncertain employment conditions during the early 1920s and early 1930s were followed by a period with nearly full employment during the 1950s and 1960s, leading to greater economic security for families and individuals (Magnusson 2000). The GDP per capita increased substantially over the study period (Statistics Sweden 2015).

The mean temperatures in Stockholm increased from 6.0°C during 1900–1929 to 7.4°C during 1980–2009 (SMHI 2013). We observed a trend of increasing MMT over the 20th century in Stockholm, Sweden. Our results suggest that the MMT could continue to rise with increasing temperatures; however, changes in personal and social determinants of health may alter this trend in the future. Our results for the end of the study period are similar to those of a recent study in which the MMT in Stockholm, Sweden, was reported to be in the 93rd percentile for the period 1990–2002 (Gasparrini et al. 2015).

Our results contradict those of a previous study of the MMT and its evolution over time, in which Miron et al. (2008) concluded that the maximum temperature of minimum mortality decreased significantly in Toledo, Spain, during 1975–2003. The authors attributed this decline to an increasing effect of heat on mortality and argued that it was due to an increasing percentage of the population being elderly and therefore, on a population level, more susceptible to mortality during heat waves. Our results are in accord with those of a recently published study of the evolution of MMT over time, in which Todd and Valleron (2015) found that the MMT increased in France during 1968–2009. The authors suggested partial adaptation to increasing temperatures as one potential explanation for their findings.

Mortality during the winter months is mostly caused by cardiovascular disease (e.g., myocardial infarction) and respiratory disease (Ebi and Mills 2013). The balance between winter and summer deaths may be altered by a changing climate; however, one review concluded that winter mortality rates are unlikely to decrease significantly as a result of warming (Ebi and Mills 2013). Staddon et al. (2014) concluded that there is no evidence that excess winter deaths in England and Wales will decrease as a result of warmer winters. A previous study in Stockholm reported that high levels of winter mortality reduced the estimated effect of high temperatures on mortality during the following summer (Rocklöv et al. 2009). Similar results were reported in Rome, Italy, a region with a warmer climate than that of Stockholm (Stafoggia et al. 2009). High levels of winter mortality will deplete the pool of susceptible individuals, likely negatively affecting the MMT because more people will die from lower hot temperatures or at less-extreme heat. Recently, Hajat et al. (2014) reached a similar conclusion when projecting future temperature-related mortality in the United Kingdom, where the burdens of cold temperatures were greater than the burden of hot temperatures for all periods, with the elderly most at risk.

The full range of the temperature–mortality relationship must be considered when evaluating the health impacts of any change in the MMT. The slopes of cold- and heat-related mortality differ, and at the extremes of the temperature range, heat generally has a much steeper slope than cold; furthermore, these slopes differ by region (Rocklöv and Ebi 2012). The estimates of MMT derived in our study depended on the number of years included in the study period; therefore, the derived MMT for a specific region might change with increased data availability. Furthermore, our derived values would likely change to some extent if the nonlinear temperature–mortality relationship were modeled differently, or if the degrees of freedom were changed. We did not explore whether there have been any changes in the temperature–mortality relationship, a relationship that is likely to have changed over time. Therefore, the results should be interpreted with some caution because the effects of temperature on health differ with the slopes of the temperature–mortality relationship. A steeper slope from the MMT for heat or cold would have more severe impacts on health than a shallower slope, even with identical values for the MMT.

If the absolute value of the MMT were fixed and all other factors were held constant, climate change (warming temperatures) would shift the relative MMT to a lower percentile of the temperature distribution. Similarly, if the relative MMT were fixed at a specific percentile of any current or future temperature distribution, climate warming would tend to increase the absolute value of the MMT. Our trend estimates based on independent 10-year periods suggest that assuming a constant value for the relative MMT (i.e., assuming that the MMT is fixed at a specific percentile of the temperature distribution regardless of changes in the distribution over time) may not be appropriate for projecting future impacts because the relative MMT may not be a valid proxy for the absolute MMT in the future. However, the use of this assumption was not supported to the same extent using independent 20-year periods, suggesting a need for more research.

Extreme high temperatures are increasing in frequency and intensity, which in turn is increasing heat-related mortality (Smith et al. 2014); however, it is reasonable to expect that people and societies can, over time, adapt to gradual increases in average temperatures, up to some limit. Our study uses historical data, making it difficult to separate adaptation resulting from co-benefits from the large medical, epidemiological, and societal changes occurring in Sweden over the 20th century from planned actions to counter the effects of gradually increasing temperatures. For Stockholm, Sweden, the association between extreme hot temperatures and short-term mortality decreased during 1901–2009 (Åström et al. 2013). A recent study in the United States reported that, over time, heat-related mortality rates for people ≥ 75 years old are declining toward rates for the 65- to 74-year-old age group, suggesting adaptation to elevated temperatures (Bobb et al. 2014). In Sweden, public awareness of the negative impacts on public health caused by elevated temperatures is, in general, low. During 1980–2009, there was limited adaptation to the number of temperature extremes occurring on a yearly basis (Oudin Åström et al. 2013). Furthermore, in a cold climate like that of Sweden, we suspect that the increased capacity of the society to cope with cold exposures through widespread and efficient indoor heating may have contributed to lowering the MMT threshold.

More research is needed on the evolution of MMT over time because it may not be appropriate to assume an increasing trend in absolute MMT, or that MMT is fixed at a specific percentile of the temperature distribution, when projecting future temperature-related mortality. Improved understanding of the evolution of MMT could inform projections of the likely future burdens of heat- and cold-related mortality over the coming decades.

## Conclusion

Our finding that the MMT has increased over time in Stockholm, Sweden, could inform projections of future impacts of climate change on public health, addressing concerns that projections may have overestimated risks by not considering adaptation. In addition to including changes in MMT, projections should also consider other drivers of temperature-related mortality, such as demographic, epidemiological, societal, and behavioral changes in human populations.
